# Effects of dysregulated glucose metabolism on the occurrence and ART outcome of endometriosis

**DOI:** 10.1186/s40001-023-01280-7

**Published:** 2023-08-30

**Authors:** Jian-Peng Chen, Yan-Ye Zhang, Jia-Ni Jin, Yue Ying, Zhi-Min Song, Qi-Qi Xu, Mi-Xue Tu, Xiao-Hang Ye, Huan-Na Tang, Fei-Da Ni, Yan-Yun Ying, Jing-Yi Li, Dan Zhang

**Affiliations:** 1 Key Laboratory of Reproductive Genetics (Ministry of Education) and Department of Reproductive Endocrinology, Women’s Hospital, Zhejiang University School of Medicine, Hangzhou, Zhejiang 310006 People’s Republic of China; 2 Zhejiang Provincial Clinical Research Center for Child Health, Women’s Hospital, Zhejiang University School of Medicine, Hangzhou, China

**Keywords:** Endometriosis, Metabolic indicator, Number of retrieved oocytes, ART outcomes, GDM

## Abstract

**Background:**

Endometriosis is associated with systemic metabolic indicators, including body mass index (BMI), glucose metabolism and lipid metabolism, while the association between metabolic indexes and the occurrence and assisted reproductive technology (ART) outcome of endometriosis is unclear. We aimed to evaluate the characteristics of systemic metabolic indexes of endometriosis patients with infertility and their effects on pregnancy outcome after ART treatment.

**Methods:**

A retrospective cohort study involve 412 endometriosis patients and 1551 controls was conducted. Primary outcome was metabolic indexes, and secondary measures consisted of the influence of metabolic indexes on the number of retrieved oocytes and ART outcomes.

**Results:**

Endometriosis patients had higher insulin (INS) [6.90(5.10–9.50) vs 6.50(4.80–8.90) μU/mL, *P* = 0.005]. A prediction model for endometriosis combining the number of previous pregnancies, CA125, fasting blood glucose (Glu) and INS, had a sensitivity of 73.9%, specificity of 67.8% and area under curve (AUC) of 0.77. There were no significant differences in ART outcomes and complications during pregnancy. The serum levels of Glu before pregnancy were associated with GDM both in endometriosis group (aOR 12.95, 95% CI 1.69–99.42, *P* = 0.014) and in control group (aOR 4.15, 95% CI 1.50–11.53, P = 0.006).

**Conclusions:**

We found serum Glu is related to the number of retrieved oocytes in control group, serum INS is related to the number of retrieved oocytes in endometriosis group, while serum Glu and INS before pregnancy are related to the occurrence of GDM in two groups. A prediction model based on metabolic indexes was established, representing a promising non-invasive method to predict endometriosis patients with known pregnancy history.

**Supplementary Information:**

The online version contains supplementary material available at 10.1186/s40001-023-01280-7.

## Background

Endometriosis is defined as the presence of active endometrial tissue outside the uterus, including endometrial glands and stroma, which can cause symptoms such as dysmenorrhea, abnormal menstruation, dyspareunia and infertility [1, 2]. The global incidence of endometriosis in women of reproductive age is about 10% and the incidence in infertile women is as high as 5 to 50% [3, 4]. As a hormone-dependent and chronic disease, endometriosis can also affect the systemic metabolic indicators, including BMI, glucose metabolism and lipid metabolism [5, 6], while the specific effects are still controversial. Metabolic indicators related to atherosclerosis have also been proved to be related to endometriosis [7, 8]. The inflammatory response in endometriosis patients can affect the metabolism of Glu and lipids which may be used as a detection method to assist in the diagnosis of endometriosis [9].

A number of women with endometriosis use ART to achieve pregnancy. But consensus is lacking on the effects of endometriosis on outcome of assisted reproduction. Some studies have found that endometriosis patients had poor ART outcomes due to factors such as decreased oocyte quality and fertilized egg quality, defective corpus luteum function, and poor endometrial receptivity [10, 11]. Other meta-analytic studies have reported there were no significant difference in ART outcome in endometriosis patients compared with patients with tubular infertility [12, 13]. Besides, metabolic indicators may be associated with ART outcome [14]. While the pathogenesis of endometriosis is closely related to metabolic factors, whether the effect of endometriosis on ART outcome is partly due to abnormal metabolic indicators is still unknown. Recent study has found endometriosis increases the risk of gestational diabetes [15], but the relationship between metabolic indicators before pregnancy and the occurrence of GDM in endometriosis is to be clarified.

We retrospectively analyzed the clinical information of endometriosis patients with infertility and infertility patients with only fallopian tube factors, compared the differences in serum metabolic indexes between two groups before receiving ART and their effects on the incidence of endometriosis, number of retrieved oocytes, as well as the pregnancy outcome after receiving ART and established a prediction model based on metabolic indexes and pregnancy history to evaluate the possibility of presence of endometriosis.

## Methods

### Study population

A retrospective analysis was undertaken to evaluate the characteristics of systemic metabolic indexes in endometriosis patients with infertility and their effects on pregnancy outcome after ART. All patients received in vitro fertilization (IVF) or intracytoplasmic sperm injection (ICSI) assisted fertility at the reproductive medicine center of Women’s Hospital, Zhejiang University School of Medicine between February 2019 and December 2020. Inclusion criteria were as follows: age 21–40 years; normal menstrual cycle, non-pregnancy nor lactation; women who had undergone laparoscopic evaluation to confirm the presence of endometriosis were included in the endometriosis group; women with tube factor as the only infertility factor through laparoscopic evaluation were included in control group.

To minimize the potential confounding factors, exclusion criteria were as follows: metabolic disease (thyroid related diseases, diabetes, hypertension, hyperprolactinemia, liver and kidney-related diseases), gynecological inflammation, chronic infectious diseases, immune diseases (anti-phospholipid antibody syndrome, systemic lupus erythematosus, rheumatoid arthritis), chromosomal or genetic abnormalities, polycystic ovary syndrome (PCOS), malignant tumors, unexplained infertility, male infertility or had received any hormone therapy in past six months. Besides, history of drinking was defined as a daily alcohol intake exceeding 10 g before or during pregnancy.

In total, 1963 cycles were enrolled from our medical database: 412 with endometriosis and 1551 without endometriosis (Control group). Available information on the dataset included maternal factors, paternal age, ART outcomes, pregnancy complications and neonate complications. Blood samples were drawn after an overnight fast.

### Ethics

This study was approved by the ethical review board of Women’s Hospital, Zhejiang University School of Medicine, Hangzhou, China (Ethics Lot number IRB-20200325-R).

### Outcome measures

Primary outcome was metabolic indexes including Glu, INS and lipids. Secondary measures consisted of the influence of metabolic indexes on ART outcomes including the number of retrieved oocytes, clinical pregnancy rate, live-birth rate, multiple pregnancies ratio, average birth weight, miscarriages and ectopic pregnancy rate, as well as the correlation between blood glucose and GDM.

We defined the ART outcome indexes included in our study based on “International Committee for Monitoring Assisted Reproductive Technology (ICMART) and the World Health Organization (WHO) Revised Glossary of ART Terminology, 2009” [16]:

### Statistical analyses

Statistical analyses were performed with Statistical Package for the Social Sciences software (SPSS version 24.0; IBM). Shapiro–Wilk test was used to assess the normality of the distribution. Normally distributed measurement data were represented by the mean ± standard deviation (x ± SD), while non-normally distributed measurement data were represented by the median (interquartile range). If the data between the two groups were normally distributed and consistent with homogeneity of variance, Student's t test was used to calculate statistical significance; otherwise, Mann–Whitney *U* nonparametric test was chosen. Rate was shown as number of cases (percentage × 100) or percentage (number of numerator cases/number of denominator cases). Differences between categorical variables were tested using Pearson’s Chi-square test. Both univariable and multivariate logistic regression models were employed to evaluate the influencing factors of endometriosis, and receiver operator control (ROC) curve was drawn. A multivariate linear regression model was used to analyze the influence of metabolic indexes on the number of retrieved oocytes. Multivariate logistic regression model was chosen to analyze the relationship between blood sugar, INS and GDM. Two-sided *P* values of less than 0.05 were considered statistically significant.

## Results

**Fig.1:**
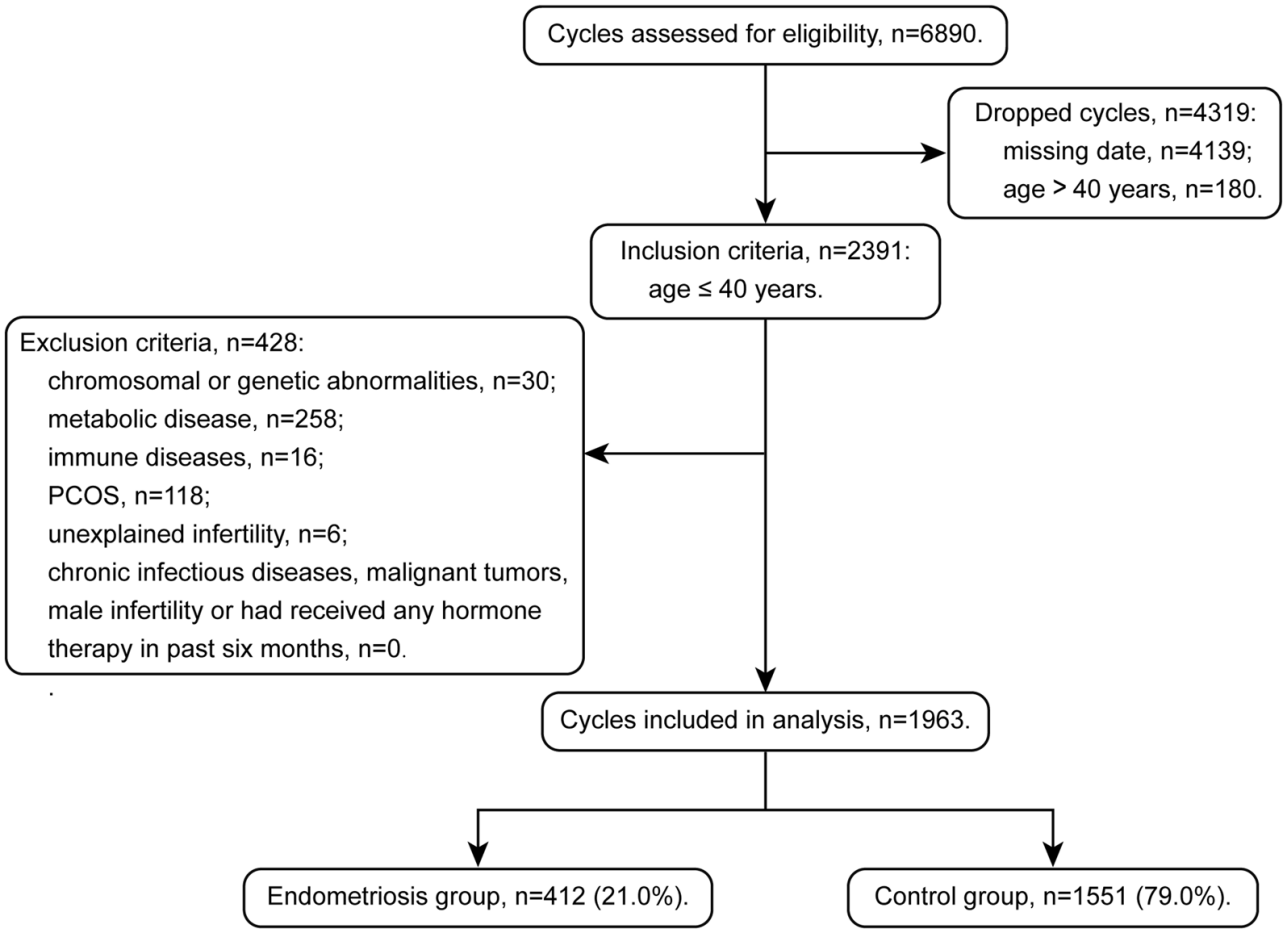
Flowchart of context diagram in the study. *PCOS* polycystic ovary syndrome

Totally, 2571 cycles were assessed for eligibility during February 2019 and December 2020 (Fig. 1). We dropped those with age over 40 (*n* = 180), chromosomal or genetic abnormalities (*n* = 30), endocrine disease or abnormal liver and kidney function (*n* = 258), immune disease (*n* = 16), PCOS (*n* = 118) and unexplained infertility (*n* = 6). Finally, 1963 cycles remained for analyses and divided into two groups, including endometriosis group (*n* = 412) and control group (*n* = 1551).

### Baseline characteristics in endometriosis patients and controls

Baseline characteristics of two study groups are summarized in Table 1. Significant differences were found for types of infertility (*P* < 0.001), history of miscarriage [116(28.2%) vs 928(59.8%), *P* < 0.001], number of previous pregnancies [0.0(0.0–1.0) vs 1.0(0.0–2.0), *P* < 0.001] and serum CA125 levels [24.00(15.10–41.20) vs 15.10(11.00–21.60) U/mL, *P* < 0.001]. Nevertheless, there were no differences in age, BMI, smoking, drinking, duration of infertility, type of ART and history of preterm delivery between two groups (*P* > 0.05, respectively).

**Table 1:** Baseline characteristics of two study groups

Characteristics	Endometriosis (*n* = 412)	Controls (*n* = 1551)	*P* value
Maternal age (years)	32 (30–35)	33 (30–36)	0.129
BMI (kg/m^2^)	21.0 (19.5–22.9)	21.3 (19.6–23.3)	0.052
Smoking			0.060
No [*n* (%)]	409 (99.3)	1518 (97.9)	
Yes [*n* (%)]	3 (0.7)	33 (2.1)	
Drinking			NA
No [*n* (%)]	412 (100.0)	1550 (99.9)	
Yes [*n* (%)]	0 (0.0)	1 (0.1)	
Duration of infertility (years)	3.0 (1.5–4.0)	2.8 (1.0–4.0)	0.248
Type of infertility			< 0.001
Primary infertility [*n* (%)]	258 (62.6)	537 (34.6)	
Secondary infertility [*n* (%)]	120 (29.1)	838 (54.0)	
Less than one year [*n* (%)]	34 (8.3)	176 (11.3)	
Type of ART			0.437
IVF [*n* (%)]	337 (79.5)	1195 (77.6)	
Half ICSI [*n* (%)]	8 (1.9)	21 (1.4)	
ICSI [*n* (%)]	79 (18.6)	323 (21.0)	
History of preterm delivery [*n* (%)]	3 (0.7)	21 (1.4)	0.438
History of miscarriage [*n* (%)]	116 (28.2)	928 (59.8)	< 0.001
Number of previous pregnancies (*n*)	0.0 (0.0–1.0)	1.0 (0.0–2.0)	< 0.001
CA125 (U/mL)	24.00 (15.10–41.20)	15.10 (11.00–21.60)	< 0.001

Values are expressed as mean ± standard deviation, median (interquartile range) or number (%)

*BMI* body mass index, *ART* assisted reproductive technology, *IVF* in vitro fertilization, *ICSI* intracytoplasmic sperm injection

### Altered serum levels of steroids and metabolic indexes in endometriosis patients

As shown in Table 2, there were no statistical differences in basal serum levels of estradiol (E_2_) and progesterone (P), Glu, triglycerides (TG), total protein (TP), alanine aminotransferase (ALT), aspartate aminotransferase (AST), creatinine, urea nitrogen, uric acid and homocysteine (HCY) between the two groups (*P* > 0.05, respectively). While we found significantly lower serum basal testosterone (T) [0.50(0.00–0.80) vs 0.60(0.00–0.90) nmol/L, *P* = 0.005], higher serum INS [6.90(5.10–9.50) vs 6.50(4.80–8.90) μU/mL, *P* = 0.005], TC [4.35(3.92–4.80) vs 4.27(3.81–4.77) mmol/L, *P* = 0.036], HDL-C [1.36(1.19–1.57) vs 1.32(1.14–1.52) mmol/L, *P* = 0.005] and LDL-C [2.63(2.17–3.01) vs 2.54(2.12–2.94) mmol/L, *P* = 0.043] in endometriosis group compared with controls.

**Table 2:** Metabolic indexes, ovarian function and ART outcomes of two study groups

Characteristics	Endometriosis (*n* = 412)	Controls (*n* = 1551)	*P* value
Basal E_2_ (pmol/L)	114.65 (67.97–156.58)	110.70 (67.70–153.60)	0.405
Basal P (nmol/L)	1.14 (0.75–1.55)	1.12 (0.76–1.50)	0.644
Basal T (nmol/L)	0.50 (0.00–0.80)	0.60 (0.00–0.90)	0.005
Glu (mmol/L)	5.01 (4.79–5.27)	5.04 (4.80–5.31)	0.134
INS (μU/mL)	6.90 (5.10–9.50)	6.50 (4.80–8.90)	0.005
TG (mmol/L)	0.92 (0.71–1.19)	0.92 (0.69–1.27)	0.435
TC (mmol/L)	4.35 (3.92–4.80)	4.27 (3.81–4.77)	0.036
HDL-C (mmol/L)	1.36 (1.19–1.57)	1.32 (1.14–1.52)	0.005
LDL-C (mmol/L)	2.63 (2.17–3.01)	2.54 (2.12–2.94)	0.043
TP (g/L)	71.97 ± 4.49	72.20 ± 4.77	0.318
ALT (U/L)	13.00 (10.00–17.00)	13.00 (10.00–18.00)	0.178
AST (U/L)	17.00 (15.00–20.00)	18.00 (15.00–20.00)	0.068
Creatinine (μmoI/L)	56.00 (50.00–62.60)	55.10 (49.00–62.00)	0.202
Urea nitrogen (mmol/L)	3.78 (3.21–4.48)	3.76 (3.18–4.51)	0.621
Uric acid (μmoI/L)	266.00 (234.25–303.00)	270.00 (231.00–312.00)	0.277
HCY (nmol/L)	9.70 (8.50–10.80)	9.80 (8.50–11.10)	0.281
AMH (ng/mL)	2.02 (1.06–3.49)	2.53 (1.48–4.07)	< 0.001
AFC (*n*)	8.00 (5.00–11.00)	10.00 (7.00–12.00)	< 0.001
Basal FSH (IU/L)	6.74 (5.26–8.35)	6.32 (4.81–7.87)	< 0.001
Basal LH (IU/L)	4.19 ± 2.40	4.60 ± 2.66	0.031
Gn dosage (IU)	2025.00 (1575.00–2700.00)	2025.00 (1575.00–2475.00)	0.664
Gn days (day)	9.00 (8.00–12.00)	9.00 (8.00–11.00)	0.058
Number of retrieved oocytes (*n*)	7.00 (4.00–11.00)	9.00 (5.00–14.00)	< 0.001
Fertilization rate [%]	64.3 (2158/3357)	64.0 (9714/15172)	0.778
Cleavage rate [%]	23.7 (512/2158)	24.1 (2338/9714)	0.736
Number of transferable embryos (*n*)	0.76 ± 0.93	0.77 ± 0.93	0.776
Number of high-quality embryos (*n*)	0.57 ± 0.82	0.60 ± 0.83	0.402
High-quality embryos rate [%]	12.1 (232/1912)	11.1 (936/8435)	0.196
Number of embryos transferred (*n*)	1.81 ± 0.39	1.78 ± 0.41	0.389
Implantation rate^1^ [%]	38.7 (122/315)	36.0 (429/1191)	0.375
Ectopic pregnancy rate^2^ [%]	2.3 (4/174)	1.5 (10/669)	0.684
Clinical pregnancy rate^3^ [%]	51.7 (90/174)	49.5 (331/669)	0.597
Miscarriage rate^4^ [%]	6.9 (12/174)	5.7 (38/669)	0.606
Delivery rate^5^ [%]	45.4 (79/174)	43.8 (293/669)	0.606
Live birth rate^6^ [%]	54.0 (94/174)	54.0 (361/669)	0.988

Values are expressed as mean ± standard deviation, median (interquartile range) or number (%)

*E*_*2*_ estradiol, *P* progesterone, *T* testosterone, *Glu* glucose, *INS* insulin, *TG* triglycerides, *TC* total cholesterol, *HDL-C* high density lipoprotein cholesterol, *LDL-C* low density lipoprotein cholesterol, *TP* total protein, *ALT* alanine aminotransferase, *AST* aspartate aminotransferase, *HCY* homocysteine, *AMH* anti-Müllerian hormone, *AFC* antral follicle counting, *FSH* follicle stimulating hormone, *LH* luteinizing hormone, *Gn* gonadotropin. 1. Implantation rate: the ratio of the number of gestational sacs to the total number of embryos transferred. 2. Ectopic pregnancy rate: the ratio of the number of Ectopic pregnancy cycles to the total number of transfer cycles. 3. Clinical pregnancy rate: the ratio of the number of clinical pregnancy cycles to the total number of transfer cycles. 4. Miscarriage rate: the ratio of the number of miscarriage cycles to the total number of transfer cycles. 5. Delivery rate: the ratio of the number of deliveries that resulted in at least one live born baby to the total number of transfer cycles. 6. Live birth rate: the ratio of the number of live born babies to the total number of live born babies

### Prediction of endometriosis by serum glu and INS

After adjusting for potential confounders, number of previous pregnancies [adjusted odds ratio (aOR) 0.51, 95% confidence interval (CI) 0.43–0.62; *P* < 0.001], serum CA125 (aOR 1.02, 95% CI 1.01–1.03; *P* < 0.001), serum Glu (aOR 0.74, 95% CI 0.56–0.97; *P* = 0.027) and serum INS (aOR 1.03, 95% CI 1.01–1.04; *P* = 0.002) were found to be significantly associated with presence of endometriosis (Table 3). Besides, compared with subjects with primary infertility, those with secondary infertility suffered from decreased incidence of endometriosis (aOR 0.70, 95% CI 0.50–0.97; *P* = 0.030). The aORs and their 95% CI were extracted and a forest plot graphic was built [17](Additional file 1: Fig. S1).

**Table 3:** Odds ratio for endometriosis in these patients

	Crude OR (95% CI)	*P* value	Adjusted OR (95% CI)	*P* value
Maternal age (years)	0.99 (0.96–1.01)	0.298	Removed	
Smoking				
No [*n* (%)]	Reference		Reference	
Yes [*n* (%)]	2.96 (0.90–9.71)	0.073	1.93 (0.48–7.74)	0.352
Number of previous pregnancies (*n*)	0.46 (0.40–0.53)	< 0.001	0.51 (0.43–0.62)	< 0.001
Type of infertility				
Primary infertility [*n* (%)]	Reference		Reference	
Secondary infertility [*n* (%)]	0.40 (0.27–0.60)	< 0.001	0.70 (0.50–0.97)	0.030
Less than one year [*n* (%)]	0.30 (0.23–0.38)	< 0.001	1.07 (0.66–1.72)	0.793
CA125 (U/mL)	1.02 (1.02–1.03)	< 0.001	1.02 (1.01–1.03)	< 0.001
AST (U/L)	1.00 (0.98–1.01)	0.589	Removed	
BMI (kg/m^2^)	0.96 (0.92–1.00)	0.058	0.99 (0.94–1.04)	0.676
Glu (mmol/L)	0.74 (0.58–0.94)	0.014	0.74 (0.56–0.97)	0.027
INS (μU/mL)	1.02 (1.00–1.03)	0.010	1.03 (1.01–1.04)	0.002
TG (mmol/L)	0.88 (0.72–1.08)	0.213	Removed	
TC (mmol/L)	1.16 (1.01–1.33)	0.031	0.95 (0.72–1.25)	0.706
HDL-C (mmol/L)	1.63 (1.14–2.35)	0.008	1.54 (0.94–2.54)	0.088
LDL-C (mmol/L)	1.16 (0.99–1.35)	0.065	1.26 (0.96–1.72)	0.137
Basal E_2_ (pmol/L)	1.00 (1.00–1.00)	0.894	Removed	
Basal P (nmol/L)	1.03 (0.97–1.09)	0.356	Removed	
Basal T (nmol/L)	0.98 (0.90–1.06)	0.595	Removed	

*OR* odds ratio, *AST* aspartate aminotransferase, *BMI* body mass index, *Glu* glucose, *INS* insulin, *TG* triglycerides, *TC* total cholesterol, *HDL-C* high density lipoprotein cholesterol, *LDL-C* low density lipoprotein cholesterol, *E*_*2*_ estradiol, *P* progesterone, *T* testosterone

**Fig. 2:**
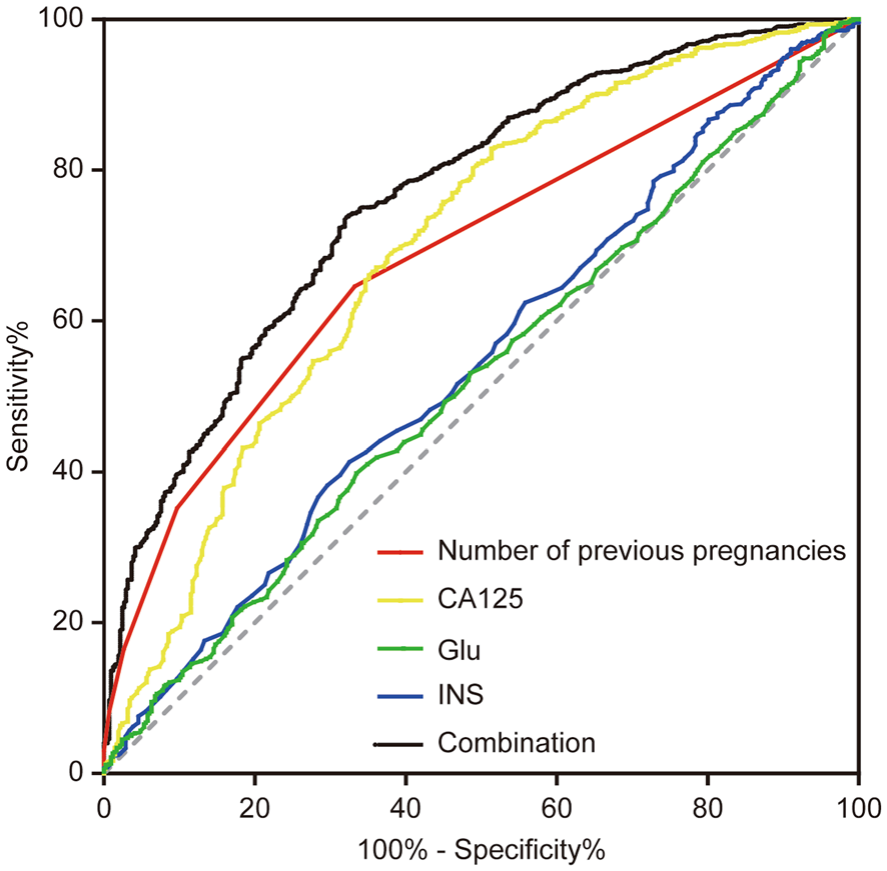
Nomogram for the prediction of endometriosis. ROC curves were produced using each potential biomarker and for the combination of them. *ROC* receiver operator control curve, *Glu* glucose, *INS* insulin

Furthermore, we performed AUC and ROC analysis to assess whether the statistically different factors found in Table 1 could be used as indicators to predict the occurrence of endometriosis [17] (Fig. 2). Results showed Glu and INS had a sensitivity of 39.9% and 41.3%, specificity of 66.5% and 67.5%, AUC of 0.52 and 0.55, respectively. When combining previous pregnancies, serum CA125, serum Glu and INS, the mode had a sensitivity of 73.9%, specificity of 67.8% and AUC of 0.77 (Additional file 2: Table S1).

### Altered serum glu and INS associated with the number of retrieved oocytes in endometriosis

As shown in Table 2, there were statistically significant differences in AMH [2.02(1.06–3.49) vs 2.53(1.48–4.07) ng/mL, *P* < 0.001], AFC [8.00(5.00–11.00) vs 10.00(7.00–12.00), *P* < 0.001], FSH [6.74(5.26–8.35) vs 6.32(4.81–7.87) IU/L, *P* < 0.001] and LH [(4.19 ± 2.40 vs 4.60 ± 2.66 IU/L, *P* = 0.031)] between endometriosis group and control group. Furthermore, the number of retrieved oocytes in endometriosis patients was significantly lower than that in control group [7.00(4.00–11.00) vs 9.00(5.00–14.00), *P* < 0.001], without differences in gonadotropin (Gn) dosage and Gn days. However, there were no statistically significant differences in fertilization rate, cleavage rate, number of transferable embryos, number of high-quality embryos, high-quality embryos rate, number of embryos transferred, implantation rate, clinical pregnancy rate, miscarriage rate, ectopic pregnancy rate, delivery rate and live-birth rate between the study groups (*P* > 0.05, respectively) (Table 2).

We further explored whether alterations in serum Glu and INS played a role in the numbers of retrieved oocytes in endometriosis by multi-factor linear regression analysis. As shown in Table 4, the number of retrieved oocytes was positively correlated with INS [0.07(0.00–0.14), *P* = 0.048] in endometriosis group. In control group, the number of retrieved oocytes was negatively correlated with Glu [− 0.80(− 1.48–− 0.12), *P* = 0.021].

**Table 4:** Multivariate logistic regression predictors of the number of retrieved oocytes

	Endometriosis (n = 412)		Controls (n = 1551)	
	β(95% CI)	*P* value	β(95% CI)	*P* value
Maternal age (years)	− 0.52 (− 0.68–− 0.31)	< 0.001	− 0.57 (− 0.64–− 0.49)	< 0.001
CA125 (U/mL)	0.00 (− 0.01–0.00)	0.201	− 0.01 (− 0.02–0.01)	0.314
Number of previous pregnancies (n)	0.95 (0.26–1.64)	0.007	0.25 (0.02–0.47)	0.032
BMI (kg/m^2^)	0.00 (− 0.23–0.154)	0.984	0.01 (− 0.11–0.14)	0.833
Basal E_2_ (pmol/L)	0.00 (0.00–0.00)	0.212	− 0.00 (0.00–0.00)	0.120
Basal P (nmol/L)	− 0.18 (− 0.43–0.07)	0.161	− 0.16 (− 0.20–0.17)	0.864
Basal T (nmol/L)	0.99 (0.37–1.60)	0.002	0.16 (− 0.04–0.36)	0.122
TG (mmol/L)	− 0.29 (− 1.24–0.67)	0.552	− 0.06 (− 0.63–0.51)	0.835
TC (mmol/L)	1.70 (0.38–3.03)	0.012	0.88 (0.10–1.67)	0.028
HDL-C (mmol/L)	− 0.83 (− 3.18–1.52)	0.490	− 0.78 (− 2.14–0.58)	0.260
LDL− C (mmol/L)	− 1.75 (− 3.19–− 0.31)	0.018	− 0.59 (− 1.43–− 0.25)	0.168
Glu (mmol/L)	0.19 (− 0.90–1.28)	0.734	− 0.80 (− 1.48–− 0.12)	0.021
INS (μU/mL)	0.07 (0.00–0.14)	0.048	− 0.03 (− 0.08–0.02)	0.264

*BMI* body mass index, *E*_*2*_ estradiol, *P* progesterone, *T* testosterone, *TG* triglycerides, *TC* total cholesterol, *HDL-C* high density lipoprotein cholesterol, *LDL-C* low density lipoprotein cholesterol, *Glu* glucose, *INS* insulin

### Neonatal outcomes and pregnancy complications in endometriosis patients and controls

Neonate outcomes in two groups are illustrated in Additional file 3: Table S2 [17]. There were no statistically significant differences in gestational week, manner of childbirth, rate of twins as well as gender and weight of both single and twin babies. As shown in Additional file 4: Table S3 [17], no significant difference was found in incidences of pregnancy complications (GDM, gestational hypertension, intra-hepatic cholestasis of pregnancy, placenta previa, placental abruption, premature rupture of membranes, umbilical cord around neck, postpartum hemorrhage, infection and hypothyroidism) or neonatal complications (neonatal respiratory distress syndrome, hypoglycemia, jaundice and infection) between endometriosis group and control group.

We further explored effects of serum Glu and INS on incidence of GDM in both groups (Table 5), and found serum Glu were significantly associated with incidence of GDM in both endometriosis group (aOR 12.95, 95% CI 1.69–99.42; P = 0.014) and control group (aOR 4.15, 95% CI 1.50–11.53; *P* = 0.006).

**Table 5:** Effects of Glu and INS before pregnancy on GDM of two study groups

	N (−/ +)	GDM (%)	Crude OR (95% CI)	*P* value	Adjusted OR (95% CI)	*P* value
Glu (mmol/L)						
Endometriosis	66/13	16.5	7.90 (1.23–50.80)	0.027	12.95 (1.69–99.42)	0.014
Controls	264/29	9.9	3.27 (1.28–8.39)	0.013	4.15 (1.50–11.53)	0.006
INS (μU/ml)						
Endometriosis	66/13	16.5	1.00 (0.93–1.07)	0.973	1.01 (0.94–1.08)	0.822
Controls	264/29	9.9	0.98 (0.89–1.08)	0.687	0.99 (0.90–1.09)	0.819

*GDM* gestational diabetes mellitus, *OR* odds ratio, *Glu* glucose, *INS* insulin

## Discussion

Metabolomics represents a useful diagnostic tool for the study of metabolic changes during a different physiological or pathological status. Clinically, endometriosis patients have abnormal metabolic manifestations, including abnormal clinical features and metabolic indexes. Recently, metabolic approach has emerged as a possible non-invasive diagnostic tool in women with or without endometriosis [18–23]. Our previous study showed that most metabolites important for glucolipid metabolism were up-regulated in follicular fluid (FF) of endometriosis patients [24]. Those data suggested dysregulated circulating metabolic molecules might play an important role in endometriosis development. We further explored whether relevant serum metabolic indexes were involved in endometriosis development via a retrospective study including 412 endometriosis patients and 1551 control patients in the present study and found endometriosis patients present with higher serum levels of INS, TC, HDL-C, LDL-C and lower serum level of basal T. By logistic regression analyses, we developed a model combining the number of previous pregnancies, serum levels of CA125, Glu and INS to predict the occurrence of endometriosis. The mode had a sensitivity of 73.9%, specificity of 67.8% and AUC of 0.77, however, further research is needed to explore the underlying mechanism.

### Glucose metabolism and endometriosis

Marianna S has confirmed that endometriosis patients had lower glucose level and higher INS level in FF [25]. Higher INS level in FF of endometriosis patients might be related to lower glucose level. Our study not only confirmed the higher INS level in endometriosis patients at serum level, but also found that serum Glu might be a protective factor for endometriosis and INS might be a risk factor. It is generally believed that glucose metabolism in endometriosis patients is increased, which explains the possible cause of low glucose in endometriosis patients [26]. Mitochondrial breathing might be impaired because of high glucose metabolism, leading to the accumulation of oxygen free radicals in the body and aggravating the occurrence and development of endometriosis. INS maintains the stability of serum Glu levels by promoting the body's intake of glucose, increasing glycogen synthesis and inhibiting gluconeogenesis and glycogen decomposition [27]. Therefore, INS within a certain range may have a benign effect on improving the ovarian function of endometriosis patients, explaining the number of retrieved oocytes is positively correlated with INS in endometriosis patients, as shown in our study. In order to investigate whether high serum INS levels in endometriosis patients were related to insulin resistance, we further calculated the HOMA index (Glu × INS/22.5) and found no significant difference between the two groups [2.50 (1.07–2.09) vs 1.53 (1.10–2.09), *P* = 0.193], indicating that the increase of insulin levels in endometriosis patients was not caused by insulin resistance. But the specific mechanism still needs further research.

### Lipid or steroid metabolism and endometriosis

In terms of lipid metabolism, Mu F found endometriosis patients were more susceptible to hypercholesterolemia and hypertension, which was most obvious among women younger than 40 years old [28]. Melo also reported that endometriosis patients had higher levels of TG, TC and LDL-C [9], consistent with our findings. While the mechanisms underlying dysregulated lipid metabolism and development of endometriosis is still unclear. Cirillo et al. have found Mediterranean dietary intervention can improve lipid or steroid metabolism in endometriosis patients [7], while it is still to be proved whether Mediterranean dietary intervention be helpful as an adjuvant treatment of endometriosis. On the other hand, many epidemiological studies reported that endometriosis women might have a lower BMI [29, 30]. But other studies found that women with a normal BMI were also likely to experience endometriosis [31, 32]. In our study, we found no difference in BMI between two groups. The diagnosis of endometriosis in our study was confirmed by laparoscopic examination, while the diagnosis of endometriosis in most previous population-based studies was just described by patients. We assume that different populations and different modes of diagnosis might also cause bias to the study results. Therefore, the association of BMI and endometriosis has yet to be confirmed.

As to steroid hormone metabolism, previous studies have found lower levels of T in endometriosis lesions [33, 34], and we further confirmed lower serum basal level of T in endometriosis patients and found the basal serum T is positively correlated with the number of retrieved oocytes in endometriosis patients (Table 3). It is generally known that the imbalance of T synthesis can lead to endometrial disease and impaired endometrial function [35], and Selak V found that danazol (17α-ethynyl testosterone) could reduce the size of endometriotic lesions [36]. Therefore, we speculated a relatively high T might be beneficial for alleviating endometriosis-related symptoms, thereby improving ovarian function and increasing the number of oocytes in endometriosis patients. Regarding the relationship between insulin and androgens, there might be a positive correlation between two indicators in PCOS, but we did not find this association in endometriosis. It is still unknown in endometriosis and further research is needed to determine.

### Metabolism dysregulation and ART outcomes of endometriosis

The incidence of infertility in endometriosis patients was higher than that of the general population, as reported by previous studies [37, 38] and also by the present study. We found the ovarian reserve and responsiveness of endometriosis patients were significantly lower, manifested by lower AMH, lower AFC, higher basal FSH, lower basal LH, and a significantly decreased number of retrieved oocytes. The impact of endometriosis on ovarian function is mainly reflected in two aspects [39]: endometriosis damages the ovary and affects ovarian function through physical compression, inflammation and blood supply; previous surgical treatment of endometriosis may also cause certain damage to the ovary. Currently, ART is the most effective treatment for endometriosis-related infertility. It is still no consensus on whether there is difference in ART outcome in infertility patients with or without endometriosis [10, 12, 13]. Several studies reported no difference in live-birth rates in subsequent IVF cycles in endometriosis patients versus tubal factor [40, 41]. Another study described lower pregnancy and live-birth rates in patients with endometrioma [9]. In our study, we found no significant difference in ART outcomes in endometriosis patients compared with the control group, although they had worse ovarian reserve and responsiveness. Similarly, several studies examining the basic morphology of oocytes and embryo development in endometriosis patients or controls have not found any differences in the two groups [42–44]. We thought the quality of the retrieved oocytes by ART in endometriosis were not much different from that of the control group and endometriosis lesion alone is unlikely to be the major contributory cause to worse reproductive outcomes, at least in the context of IVF/ICSI.

We also explored whether the dysregulated metabolic indexes had effects on the number of retrieved oocytes, ART outcome and the incidence of pregnancy complications in endometriosis patients, and we found the number of retrieved oocytes was positively correlated with INS in endometriosis group, while the number of retrieved oocytes was negatively correlated with Glu in control group. Interestingly, our results showed that serum levels of Glu were significantly associated with incidence of GDM both in endometriosis group and in control group, suggesting that the higher the blood glucose level before pregnancy, the greater the incidence of GDM during pregnancy, which might shed light on preventing the occurrence of GDM in clinical work. Some studies have found endometriosis increases the risk of gestational diabetes [15], but others have shown the opposite [45]. However, we found no significant difference in the incidence of GDM between the two groups (16.5% vs 9.9%, *P* = 0.102). We think further prospective cohort study is required to clarify this controversial association.

## Strengths and limitations

Some main strengths of this study deserve to be mentioned. We excluded patients without a definitive diagnosis of endometriosis by laparoscopy. Moreover, logistic regression analysis might have further lessened the impact of the confounders, in which we matched age, CA125, types of infertility and other baseline characteristics to protect our data from other confounders. As for limitations, endometriosis patients included in this study had a history of endometriosis-related surgery, but the control group did not though they had laparoscopic evaluation. Some studies believed that surgery could improve female fertility conditions [1], while other studies thought surgery might cause damage to the ovaries [46]. Secondly, we could not perform subgroup analyses according to disease stages in retrospective study, because endometriosis was heterogeneous, and the severity of endometriosis might directly affect ART outcomes [41]. Thirdly, some basal characteristics of the two groups differed and we could not fully exclude the influence of confounders. Finally, our study was conducted in a single reproductive medical center with standardized laboratory techniques and ART protocols, and multi-center-based randomized controlled trials are suggested in the future study.

## Conclusion

We found serum Glu is related to the number of retrieved oocytes in control group, serum INS is related to the number of retrieved oocytes in endometriosis group, while serum Glu and INS before pregnancy are related to the occurrence of GDM in two groups. We also established a prediction model based on metabolic indexes to evaluate the possibility of presence of endometriosis, which might represent a promising non-invasive method to predict endometriosis patients with known pregnancy history, but further study is warranted to verify. Our findings suggest clinicians pay more attention to serum Glu before pregnancy, which was relevant with occurrence of GDM. In conclusion, the present study shed light on the effects of dysregulated glucose metabolism on the occurrence and ART outcome of endometriosis, while the underlying mechanism is jet to be clarified.

### Supplementary Information

**Additional file 1: Fig. S1.** Forest plot of metabolic index in predicting endometriosis. *OR* odds ratio, *HDL-C* high density lipoprotein cholesterol, *INS* insulin, *TC* total cholesterol, *Glu* glucose.

**Additional file 2: Table S1.** Sensitivity and specificity of potential biomarkers for diagnosis of endometriosis.

**Additional file 3: Table S2.** Neonate outcomes of two study groups.

**Additional file 4: Table S3.** Pregnancy complications and neonate complications of two study groups.

## Data Availability

The datasets used and/or analyzed during the current study are available from the corresponding author on reasonable request. The following supporting information is available in the [Harvard Dataverse] repository and can be downloaded at: https://doi.org/10.7910/DVN/THVJUU.

## Abbreviations

AFC: Antral follicle counting
ALT: Alanine aminotransferase
AMH: Anti-müllerian hormone
ART: Assisted reproductive technology
AST: Aspartate aminotransferase
BMI: Body mass index
E2: Estradiol
FSH: Follicle stimulating hormone
GDM: Gestational diabetes mellitus
Glu: Glucose
Gn: Gonadotropin
HDL-C: High density lipoprotein cholesterol
HCY: Homocysteine
ICSI: Intracytoplasmic sperm injection
INS: Insulin
IVF: In vitro fertilization
LDL-C: Low density lipoprotein cholesterol
LH: Luteinizing hormone
OR: Odds ratio
P: Progesterone
T: Testosterone
TC: Total cholesterol
TG: Triglycerides
TP: Total protein
