# Supplementary figures and images for: Effects of dysregulated glucose metabolism on the occurrence and ART outcome of endometriosis
Source: Eur J Med Res. 2023 Aug 30;28:305. doi: 10.1186/s40001-023-01280-7 (PMC10466766; doi:10.1186/s40001-023-01280-7)

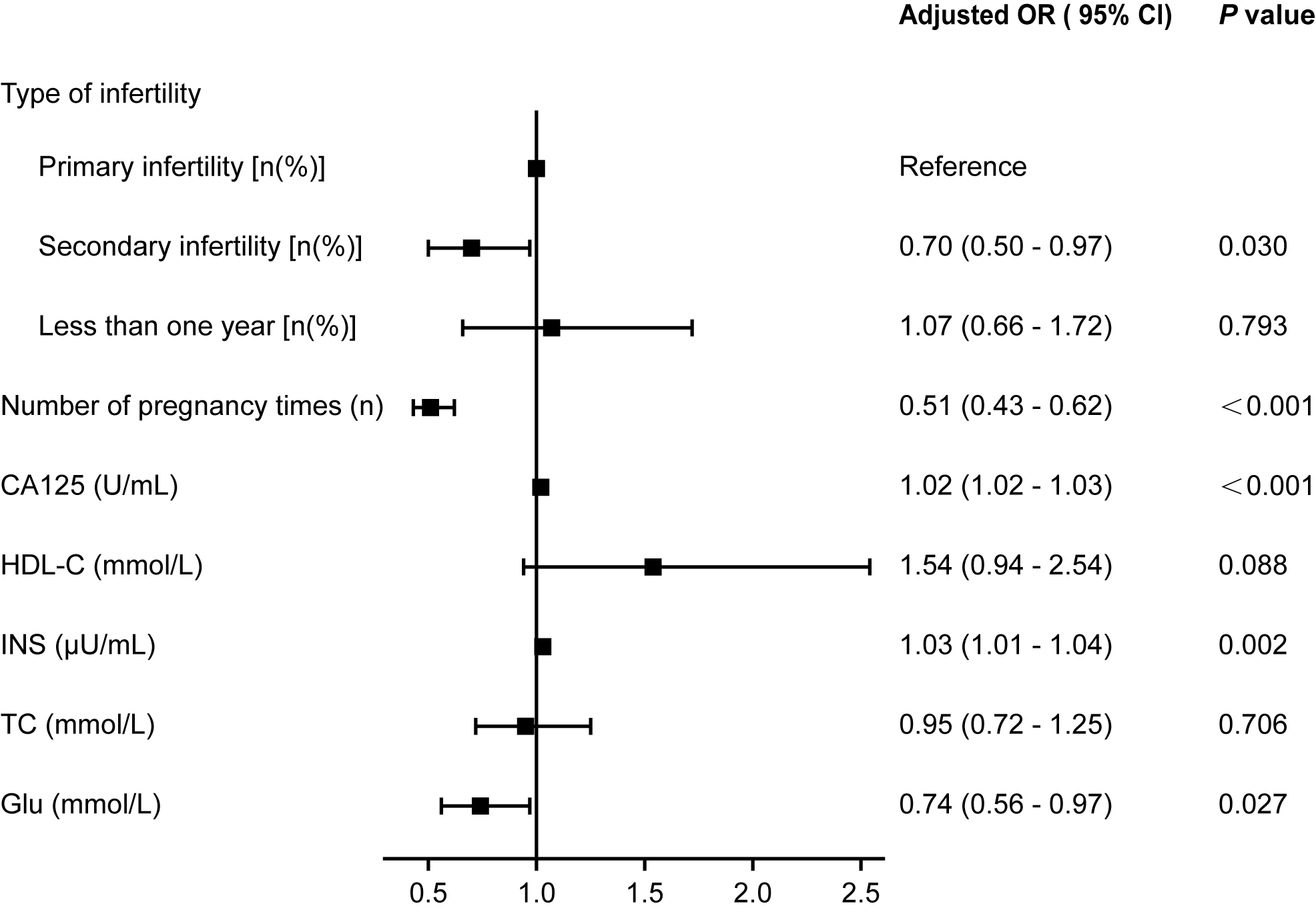

Supplement: Supplementary file 1 — Additional file 1: Fig. S1. Forest plot of metabolic index in predicting endometriosis. OR odds ratio, HDL-C high density lipoprotein cholesterol, INS insulin, TC total cholesterol, Glu glucose. [file 40001_2023_1280_MOESM1_ESM.tif]
