# Supplementary material for: Effects of dysregulated glucose metabolism on the occurrence and ART outcome of endometriosis
Source: Eur J Med Res. 2023 Aug 30;28:305. doi: 10.1186/s40001-023-01280-7 (PMC10466766; doi:10.1186/s40001-023-01280-7)
Supplement: Supplementary file 2 — Additional file 2: Table S1. Sensitivity and specificity of potential biomarkers for diagnosis of endometriosis. [file 40001_2023_1280_MOESM2_ESM.docx]

**Additional file 2: Table S1** Sensitivity and specificity of potential biomarkers for diagnosis of endometriosis

|  | **AUC** | ***P* value** | **Sensitivity (%)** | **Specificity (%)** | **Truncated**  **value** |
| --- | --- | --- | --- | --- | --- |
| Number of previous pregnancies (n) | 0.69 (0.66-0.71) | < 0.001 | 64.6 | 66.8 | 0.5 |
| CA125 (U/mL) | 0.70 (0.67-0.73) | < 0.001 | 82.8 | 48.7 | 25.1 |
| Glu (mmol/L) | 0.52 (0.49-0.55) | 0.135 | 39.9 | 66.5 | 5.15 |
| INS (μU/mL) | 0.55 (0.51-0.58) | 0.005 | 41.3 | 67.5 | 5.85 |
| Combination | 0.77 (0.74-0.79) | < 0.001 | 73.9 | 67.8 | 0.26 |

*ROC* receiver operator control curve, *Glu* glucose, *INS* insulin, *AUC* area under the curve
