# Supplementary material for: Effects of dysregulated glucose metabolism on the occurrence and ART outcome of endometriosis
Source: Eur J Med Res. 2023 Aug 30;28:305. doi: 10.1186/s40001-023-01280-7 (PMC10466766; doi:10.1186/s40001-023-01280-7)
Supplement: Supplementary file 3 — Additional file 3: Table S2. Neonate outcomes of two study groups. [file 40001_2023_1280_MOESM3_ESM.docx]

**Additional file 3: Table S2** Neonate outcomes of two study groups

|  | **Endometriosis**  **(n=79)** | **Controls**  **(n=293)** | ***P* value** |
| --- | --- | --- | --- |
| Gestational week |  |  | 0.174 |
| Preterm delivery ^1^[n (%)] | 11 (13.9) | 60 (20.5) |  |
| Full-term delivery [n (%)] | 68 (86.1) | 233 (79.5) |  |
| Manner of childbirth |  |  | 0.233 |
| Vaginal birth [n (%)] | 38 (48.1) | 121 (41.3) |  |
| Cesarean section [n (%)] | 41 (51.9) | 172 (58.7) |  |
| Number of live births |  |  |  |
| Single baby | 64 (81.0) | 225 (76.8) | 0.988 |
| Male [n (%)] | 33 (50.0) | 114 (50.7) | 0.924 |
| Female [n (%)] | 33 (50.0) | 111 (49.3) |  |
| Weight (kg) | 3.07±0.38 | 3.39±0.40 | 0.073 |
| Twin babies | 15 (19.0) | 68 (23.2) |  |
| Male [n (%)] | 15 (50.0) | 73 (53.7) | 0.715 |
| Female [n (%)] | 15 (50.0) | 63 (46.3) |  |
| Weight (kg) | 2.56±0.66 | 2.45±0.50 | 0.310 |

Values are expressed as mean ± standard deviation or percentage (number/number).

1. Preterm birth: delivered after 28 weeks but before 37 weeks.
