# Supplementary material for: Effects of dysregulated glucose metabolism on the occurrence and ART outcome of endometriosis
Source: Eur J Med Res. 2023 Aug 30;28:305. doi: 10.1186/s40001-023-01280-7 (PMC10466766; doi:10.1186/s40001-023-01280-7)
Supplement: Supplementary file 4 — Additional file 4: Table S3. Pregnancy complications and neonate complications of two study groups. [file 40001_2023_1280_MOESM4_ESM.docx]

**Additional file 4: Table S3** Pregnancy complications and neonate complications of two study groups

|  | **Endometriosis** | **Controls** | ***P* value** |
| --- | --- | --- | --- |
| Complication during pregnancy | n=79 | n=293 |  |
| GDM [n (%)] | 13 (16.5) | 29 (9.9) | 0.102 |
| Gestational hypertension [n (%)] | 2 (2.5) | 11 (3.8) | 0.857 |
| ICP [n (%)] | 2 (2.5) | 9 (3.1) | NA |
| Placenta previa [n (%)] | 1 (1.3) | 4 (1.4) | NA |
| Placental abruption [n (%)] | 1 (1.3) | 10 (3.4) | 0.532 |
| Premature rupture of membranes [n (%)] | 3 (3.8) | 9 (3.1) | NA |
| Umbilical cord around neck [n (%)] | 8 (10.1) | 15 (5.1) | 0.101 |
| Postpartum hemorrhage [n (%)] | 2 (2.5) | 2 (0.7) | 0.424 |
| Infection [n (%)] | 1 (1.3) | 2 (0.7) | NA |
| Hypothyroidism [n (%)] | 2 (2.5) | 3 (1.0) | 0.630 |
| Neonate complications | n=94 | n=457 |  |
| NRDS [n (%)] | 4 (4.3) | 13 (2.8) | 0.694 |
| Hypoglycemia [n (%)] | 1 (1.3) | 10 (2.2) | 0.760 |
| Jaundice [n (%)] | 3 (3.2) | 31 (6.8) | 0.279 |
| Infection [n (%)] | 1 (1.3) | 5 (1.1) | NA |

Values are expressed as number (%).

*GDM* gestational diabetes mellitus, *ICP* intrahepatic cholestasis of pregnancy, *NRDS* neonatal respiratory distress syndrome
